# Supplementary figures and images for: The proteolysis of ZP proteins is essential to control cell membrane structure and integrity of developing tracheal tubes in Drosophila
Source: eLife. 2023 Oct 24;12:e91079. doi: 10.7554/eLife.91079 (PMC10597583; doi:10.7554/eLife.91079)

Figure 5D

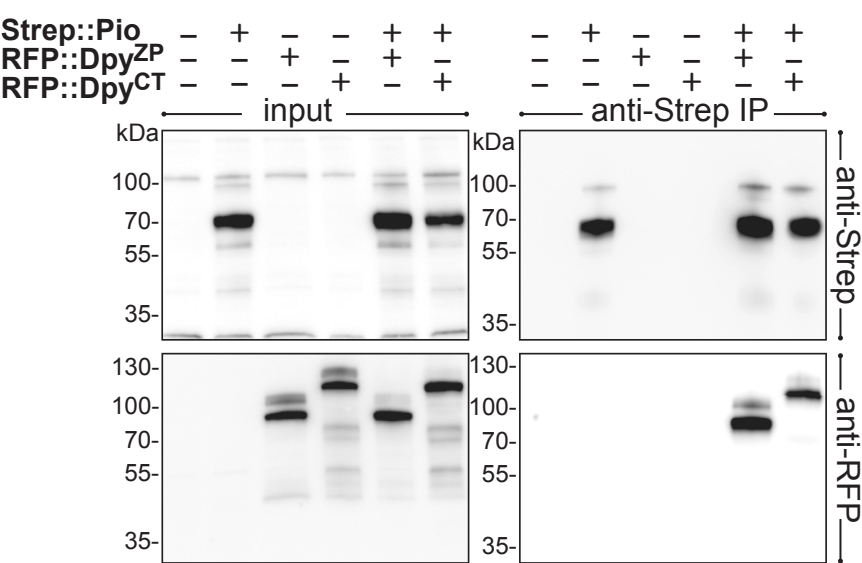

Crops of original blots

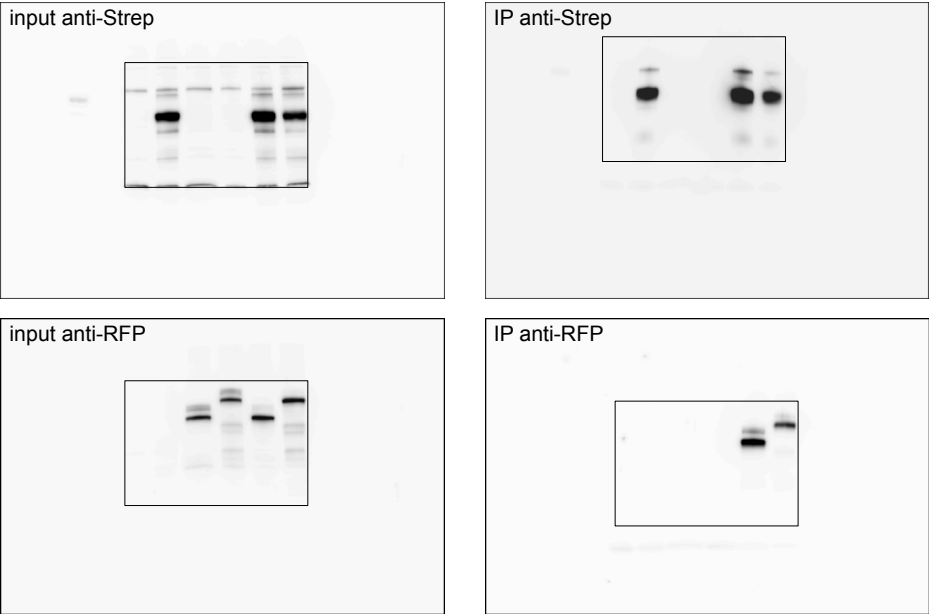

Supplement: Figure 5—source data 7. [file elife-91079-fig5-data7.pdf]

Figure 6A

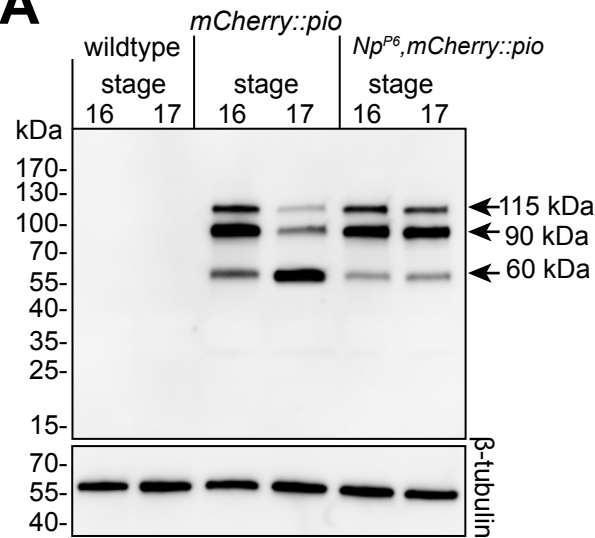

Crops of original blots

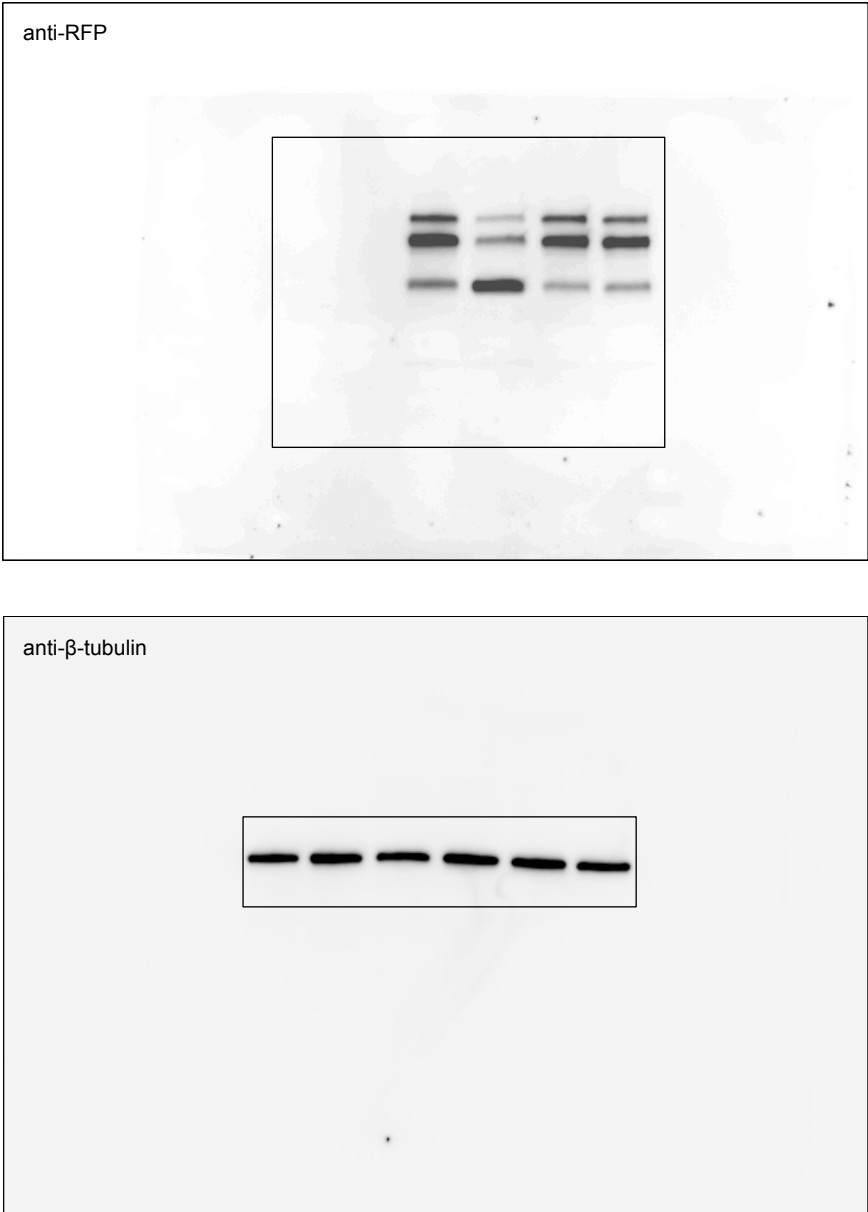

Supplement: Figure 6—source data 7. [file elife-91079-fig6-data7.pdf]

Figure 6B

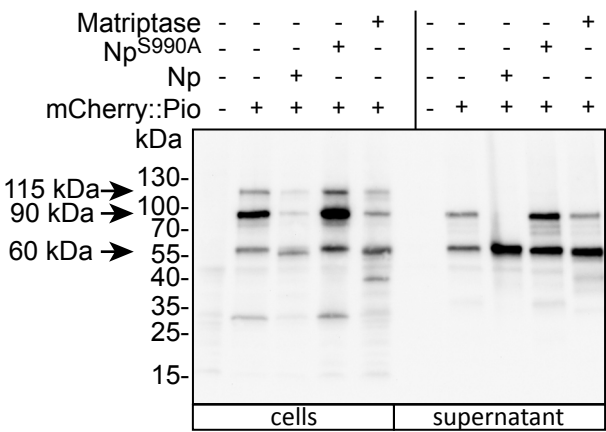

Crop of original blot

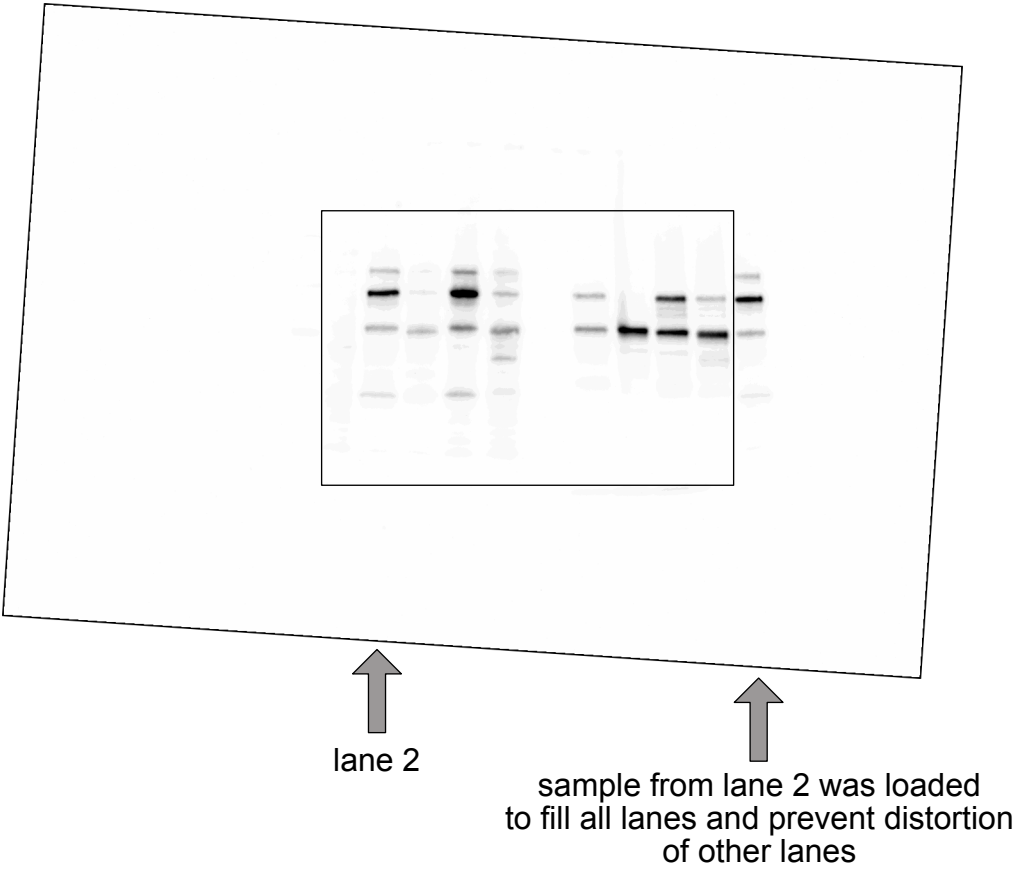

Supplement: Figure 6—source data 8. [file elife-91079-fig6-data8.pdf]
